# Next generation sequencing: clinical applications in solid tumours

**DOI:** 10.1007/s12254-017-0361-1

**Published:** 2017-11-03

**Authors:** Leonhard Müllauer

**Affiliations:** Department of Pathology, Medical University Vienna, Waehringer Guertel 18–20, 1090 Vienna, Austria

**Keywords:** Next generation sequencing, Tumor genetics, Molecular pathology, Liquid biopsy, Personalized medicine

## Abstract

Next generation sequencing (NGS) has unravelled the genetic alterations that underlie the pathogenesis of cancer. It is now becoming integrated into routine clinical diagnostics of malignant tumours. NGS supports diagnosis, identifies therapeutic targets, reveals resistance mechanisms and facilitates disease monitoring. It takes a central function in the implementation of cancer therapies adapted to the molecular alterations of tumours.

Frederik Sanger and Walter Gilbert developed techniques to determine the nucleotide sequence of DNA in the 1970s [1, 2]. In the years to follow the Sanger technique became prevalent because of its simplicity [3]. The sequencing reaction products were labelled with radionucleotides, separated on large vertical polyacrylamide gels and visualised by autoradiography. In the 1990s, Sanger sequencing was partially automated, and the separation and autoradiography steps were replaced by tiny capillary gel electrophoresis and laser*-*induced fluorescence detection of reaction products. Currently, the most advanced capillary gel electrophoresis sequencer achieves a maximum output of 1.6 megabases per day.

About ten years ago, new sequencing technologies emerged which are termed “next generation sequencing” (NGS) [3–6]. In comparison to Sanger sequencing, NGS exhibits a massively increased output, ranging from a few gigabases per run with small benchtop sequencers to 6000 gigabases with large instruments. With NGS the simultaneous sequencing of millions of different DNA molecules is possible. Furthermore, compared to Sanger sequencing, NGS is more versatile. It also provides for the detection of gene amplifications and deletions and the determination of gene expression. Moreover, the method is widely scalable. It may comprise variably sized gene panels, the whole human exome (≈30 megabases), genome (≈3.3 gigabases) or even the entire human transcriptome. And the tagging with unique molecular barcodes allows for the mixing and simultaneous sequencing of samples from different patients.

NGS has dramatically reduced the cost per base of sequencing while increasing the speed of sequencing. The first sequencing of the human genome with the Sanger technique took about 13 years [7]. With NGS, the sequencing of a human genome is now possible within one week and the costs with high throughput sequencers have meanwhile approached the magical limit of 1000 US dollars [8].

A central characteristic of NGS is the spatial separation of individual DNA molecules, which facilitates the simultaneous sequencing of millions of different stretches of DNA. The DNA molecules in the two currently prevailing platforms are either fixed, amplified and sequenced on tiny glass plates (Illumina^**©**^ [San Diego, CA, USA]), or else separated and amplified in lipid droplets and then distributed to millions of miniature wells on tiny semiconductor chips (Ion Torrent^©^ [Thermo Fisher, Waltham, MA, USA]) [3–6].

The pool of individual DNA molecules to be sequenced is called the “library”. The library can for example encompass only a few genes, all RNA transcripts or the whole genome. A gene panel library is generated by selective amplification of the target regions with multiplexed polymerase chain reactions (PCRs) or by hybridisation capture with DNA baits (Fig. 1; [9, 10]). Hybridisation capture may utilise biotinylated oligonucleotide probes specific for target regions of interest that are hybridised to DNA in solution. Next, the biotinylated probe-target hybrids are isolated by streptavidin-coated magnetic beads to obtain libraries enriched for the target regions.

**Fig. 1:**
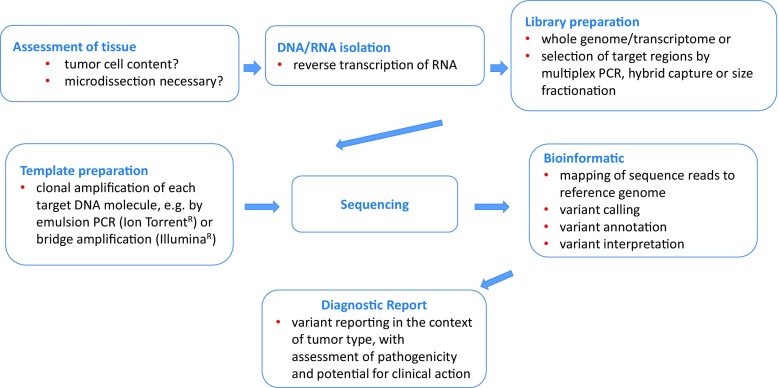
The process of next generation sequencing of tumour tissue. *PCR* polymerase chain reaction

Next generation sequencing has enabled national and international consortia to perform the comprehensive identification of the genetic aberrations in tumours. The Cancer Genome Atlas (TCGA; http://cancergenome.nih.gov/) and the International Cancer Genome Consortium (ICGC; https://icgc.org/icgc) have analysed the exomes, transcriptomes and epigenomes for tens of thousands of tumours of different entities. The genomic datasets are freely available to the public (https://portal.gdc.cancer.gov/ and https://dcc.icgc.org/). Most solid tumours exhibit between 40 and 150 non-silent mutations that predict a modification of the amino acid sequences of the encoded proteins [11]. However, the number of driver mutations that exert an effect on the pathogenesis of a tumour is limited. The majority of the mutations are regarded as passenger mutations that do not contribute to the malignant phenotype [11, 12]. Most genes are rarely mutated, in the low one-digit percent range; only a few genes, such as TP53 or KRAS, are frequently mutated in various tumour entities. The low frequency of most mutations poses a challenge to the development of targeted drugs and clinical studies and has spurred the design of basket and umbrella trials, in which targeted therapy is tailored primarily to the molecular alteration and not to the histological type [13].

The determination of a tumour’s genetic aberrations by NGS can provide decisive information on the histopathological classification, reveal targets for therapy, indicate drug resistance and give prognostic information. Currently, the sequencing of gene panels that interrogates a few to the low hundreds of genes prevails. Advantages of panel sequencing in comparison to whole exome or whole genome sequencing include lower costs, easier bioinformatic interpretation, faster sample throughput and lower data storage requirements. Furthermore, gene panels achieve a higher coverage of sequencing, i. e. the number of times a particular position in the DNA is sequenced [14]. In general, a higher coverage results in a higher sensitivity and accuracy of mutation detection. Additionally, the number of genetic aberrations with an associated available approved drug is still very limited, and this small number of genetic targets can be covered by panel sequencing. However, if in clinical trials NGS results are used to stratify patients, then the analysis of a larger number of genes may become necessary.

Tumour tissue for NGS should be evaluated by a clinical pathologist. It is crucial to assign the correct diagnosis, identify the tumour cells on histological slides and exclude necrotic areas. In case of focal infiltration, the tumour area should be marked for microdissection to enrich the tumour cells for DNA and RNA extraction and thereby enhance the sensitivity of mutation detection. In addition, the fraction of tumour cell nuclei in the tissue must be estimated. This constitutes an important piece of information for the interpretation of the frequency of mutated alleles, e. g. a mutated allele frequency of only 5% but a tumour cell content of 80% indicates a subclonal mutation.

DNA and RNA isolated from native, fresh-frozen tissue as well as formalin-fixed and paraffin-embedded (FFPE) tissue are suitable for NGS. However, DNA and RNA from FFPE tissue are of inferior quality due to fragmentation and chemical modifications induced by formalin, which may result in increased false-positive and -negative mutation calls [15, 16]. Nevertheless, FFPE-derived nucleic acids are acceptable for most targeted gene panel sequencing protocols. Recently, methods have been refined to accomplish also exome and whole genome sequencing with FFPE tissue-derived DNA [17, 18]. However, for clinical diagnostics fresh-frozen tissue remains the better and preferred source for exome and, in particular, whole genome and transcriptome sequencing.

Ideally, tumour DNA sequencing is performed in parallel with the patient’s normal cell DNA, e. g. derived from blood, buccal swabs or tissue, to differentiate mutations from normal variants (polymorphisms). However, this approach doubles the costs, and the analysis of germline DNA may raise legal issues regarding stringent data protection, informed consent and genetic counselling, due to the incidental detection of hereditary pathogenic mutations. NGS reads are usually mapped to a human reference genome (https://www.ncbi.nlm.nih.gov/grc) and to reference sequences deposited e. g. in the NCBI (https://www.ncbi.nlm.nih.gov/) or EMBL-EBI (http://www.ensembl.org/index.html) database. The decision as to whether a sequence difference between tumour DNA and a reference sequence is a mutation or a variant (polymorphism) may prove difficult to make. As for differentiation, databases that comprise large numbers of germline variants (polymorphisms), such as the datasets of dbSNP (http://www.ncbi.nlm.nih.gov/projects/SNP/), the 1000 Genomes Consortium (http://www.internationalgenome.org/) [19] or the Exome Aggregation Consortium (ExAC; http://exac.broadinstitute.org/), are helpful.

A further central question to address is whether a mutation has an effect on the malignant phenotype and thus represents a driver mutation or constitutes a non-pathogenic passenger mutation [11, 20]. The frequency of the mutation in numerous tumours in comparison to other genes is a parameter for the assessment of pathogenicity. Furthermore, biophysical calculations of the most likely effect of the mutation on the coding protein and the conservation of the mutated amino acid residue in different species are utilised. Additionally, results from functional assays, such as the effect of overexpression or knockout of the mutated gene in cell cultures and genetically modified animals, are taken into account. Moreover, commercially available software tools and databases are used increasingly to compare the patients’ tumour variants to the current state of knowledge on drug response and available clinical trials.

NGS is also increasingly utilised for liquid biopsies—the analysis of tumour cell-derived DNA that circulates in plasma (ctDNA) [21, 22]. The largest challenge faced in the analysis of ctDNA is the mostly low frequency of mutated alleles in the whole free-circulating plasma DNA (cfDNA) which contains also DNA released from normal cells. In extreme cases, such as an early tumour stage, the ctDNA fraction may be below 0.01% of the cfDNA. The currently prevalent methods of mutation detection in ctDNA are mutation-specific PCR and NGS. For PCR, methods with a particularly high sensitivity are necessary, such as droplet digital PCR [23]. The use of NGS for liquid biopsies requires modifications of standard protocols. The error rate of NGS lies, depending on the platform, between 0.1 and 1%. Therefore, a mutation with an allele frequency below that threshold cannot be reliably differentiated from background noise. NGS protocols have been developed that reduce the error rate, e. g. by incorporating unique molecular identifiers prior to PCR amplification of the DNA molecules [24]. Recently, vendors of NGS equipment have adopted such techniques in their product portfolio and thus made it easier for clinical diagnostic laboratories to employ these protocols, which has led to a recent increase in gene panel sequencing for liquid biopsies. With this rapid improvement of liquid biopsy detection techniques, a sensitivity of more than 80% and a specificity of 98–100% have been achieved in recent reports [25]. The at present best-established indication for liquid biopsy in oncology is the detection of the EGFR T790M resistance mutation [26]. It emerges in approximately 60% of tyrosine kinase inhibitor-treated lung adenocarcinomas with an initially sensitising non-T790M EGFR mutation. Tumours showing the T790M resistance mutation may respond to the third-generation EGFR inhibitor osimertinib. With the use of liquid biopsy, approximately 70% of patients can forego biopsy for T790M interrogation. However, those with a T790M-negative liquid biopsy still need to undergo tumour biopsy to determine the presence or absence of T790M.

In the near future more comprehensive diagnostic pan-cancer panels that simultaneously interrogate mutations, gene copy numbers and translocations will be implemented [27]. Furthermore, exome and transcriptome sequencing will become more frequent. With more extensive sequencing, the likelihood of incidentally detecting genetic aberrations that predispose an individual to an illness different from the actually interrogated neoplastic disease will increase. At present, there is a lack of consistency in the reporting of such incidental findings, which clearly calls for the establishment of precise guidelines [28, 29]. NGS may additionally facilitate the widespread screening for hereditary cancer syndromes and enhance the potential of liquid biopsies. The extensive genetic tumour profiling with current NGS and emerging third-generation sequencing technologies [3, 5, 6, 30] will become the standard of care.
